# The link between vision impairment and depressive symptomatology in late life: does having a partner matter?

**DOI:** 10.1007/s10433-021-00653-3

**Published:** 2021-09-29

**Authors:** Anna Amilon, Anu Siren

**Affiliations:** 1 The Danish Center for Social Science Research, Copenhagen, Denmark; 2 Faculty of Social Sciences (Health Sciences) and Gerontology Research Center, Tampere University, Tampere, Finland

**Keywords:** Vision impairment, Depressive symptoms, Older adults, Partnership status, Activities of daily living, Emotional support

## Abstract

Visual impairment contributes to poor mental health among older adults by restricting everyday functioning and participation. This study examined whether the negative link between vision impairment and depressive symptomatology was less severe among partnered than among single older adults. We merged data from a survey among people with vision impairment with a reference population from the most recent wave of the Danish Longitudinal Study of Ageing (DLSA) (*N* = 5831 *M*_*age*_ = 74.37, range: 65–97 years, 53.1% female), investigating whether paths from poor vision via three mediators—functional limitations, emotional support and participation in social activities—to depressive symptomatology differ by partnership status. Structural equation modeling suggested that the direct path from vision impairment to depressive symptomatology is more than twice as strong for single than for partnered older adults. Thus being partnered reduces the negative link from vision impairment to depressive symptomatology. However, the path from vision impairment to emotional support is significantly stronger among single than among partnered individuals. Thus negative spillover effects from the visual impairment on the non-impaired partner’s mental health may compromise that partner’s ability to provide emotional support. Taking into account both partnership status and the mental health of both partners may help professionals more precisely target interventions aimed at reducing the risk of depression in visually impaired older adults.

## Introduction

Globally, one in four of those aged 70 or above suffers from vision impairment (Stevens et al., 2013), and as populations age prevalence will likely increase substantially in the coming decades (Bourne et al., 2017). Vision impairment has a negative impact on both physical functioning and the ability to perform everyday tasks and activities such as mobility, reading, driving, meal preparation, shopping, housecleaning and self-care (Hajek et al., 2016; Taylor et al., 2016). Moreover, vision impairment is a strong predictor of depression, or depressive symptomatology, among older adults (Bookwala & Lawson, 2011; Carrière et al., 2013; Gong et al., 2020; Kempen et al., 2014). Path analysis has shown that vision impairment contributes to depressive symptomatology both directly and indirectly, through increased functional limitations and social isolation (Bookwala & Lawson, 2011; van Nispen et al., 2016).

People with vision impairment have a higher probability of experiencing difficulties performing activities of daily living (Gopinath et al., 2014; Hajek et al., 2016; Hochberg et al., 2012; Kempen et al., 2014; O’Donnell, 2005; Taylor et al., 2020; van Nispen et al., 2016), and functional limitations in activities of daily living are associated with depressive symptomatology. While some studies have demonstrated that functional limitations have a mediating role between vision impairment and depressive symptoms (Gong et al., 2020; Kempen et al., 2014), other studies have found that, after correcting for functional limitations in activities of daily living, the association between vision impairment and depressive symptomatology is no longer significant (Evans et al., 2007; van Nispen et al., 2016).

Social isolation related to vision impairment may also lead to depressive mood. Studies have found increasing avoidance of social situations among older adults with vision impairment (Bookwala & Lawson, 2011), and visual limitations in late life are associated with losses in social interactions (O’Donnell, 2005). Social withdrawal and restricted ability to participate in pleasurable activities and hobbies may contribute to the association between visual impairment and depression (Cosh et al., 2019).

While previous studies have analyzed the direct and indirect links between vision impairment, potential mediators and symptoms of depression, they have done so without taking into account potential path differences according to partnership status (Bookwala & Lawson, 2011; Gong et al., 2020). The aim of this study was to fill this gap in the literature by separately analyzing both direct and indirect links between vision impairment and depressive symptomatology among partnered and single older adults.

### The importance of a partner

Numerous studies have demonstrated the benefits of marriage or similar partnership for health and quality of life among older adults (Brown & Wright, 2017; Siren et al., 2018), and spouses provide various types of practical, financial, emotional and social support to each other (AARP and National Alliance for Caregiving, 2020; Kim et al., 2016; Lehane et al., 2017; Lehane, et al., 2018a, b). A spouse not only provides direct support and help, but also increases the availability of other social resources: Having a partner is associated with the probability of having children, and the availability of social ties (both directly and in combination with parental status) (Dykstra & Hagestad, 2007). For older adults with vision impairment, the positive effects of a partnership may be even more significant. Practical help and support from a spouse can alleviate the functional limitations due to visual impairment and social and emotional support received from a spouse can compensate for losses in other social relations due to impairment.

Studies have found that spouses provide various forms of support in coping with sensory loss (Alma et al., 2011; Lehane, et al., 2018a, b) and that cohabiting with a partner is beneficial for overall mental health among visually impaired older adults (Alma et al., 2011; Kempen et al., 2014; van Nispen et al., 2016). However, the potential interaction effect between vision impairment and partnership status, i.e., whether visually impaired older adults gain stronger positive effect from having a spouse, remains largely unknown (Alma et al., 2011; Kempen et al., 2014). Thus far, only one study has investigated this interaction, and found that although having a partner reduces the risk of depression, this association is not influenced by vision impairment (Van Nispen et al., 2016).

Furthermore, systematic knowledge on the relationships between partnership status and depressive symptomatology among people with vision impairment is scarce. The ways and situations in which having a partner may or may not protect from depressive symptoms in vision impairment remain largely unknown. Social support theory suggests that social relations help individuals’ adapt to life stressors (Cohen, 2004), but evidence on vision impairment is scarce and inconclusive. Some studies have demonstrated perceived social support to be protective for psychological well-being among older adults with dual-sensory loss (Lehane et al., 2017; Lehane, et al., 2018a, b), but Van Nispen et al. (2016) found no mediating effect of the amount of emotional support received and depressive symptomatology among people with vision impairment. Kempen et al. (2014) found that social support suppressed the (negative) relationship between low vision and depressive symptoms—a result they explain as social support being associated positively with low vision but negatively with depression.

Marital quality has also been suggested to influence the links between visual impairment, depressive symptoms and partnership status (Bookwala, 2011). However, disentangling the causal relationship between marital quality and depressive symptomatology among people with vision impairment is difficult. Spouses’ perception of marital quality is influenced by both individual and spousal health (Wong & Waite, 2015), and for vision loss, studies have found negative spillover effects of one spouse’s impairment on the other’s mental health (Lehane et al., 2017; Strawbridge et al., 2007).

### The present study

This study investigates the role of three factors that may mediate the relationship between vision impairment and depressive symptomatology among older adults: functional limitations, emotional support and participation in activities. We expect visual impairment to be positively associated with depressive symptomatology, both directly and indirectly, with functional limitations, emotional support and participation in activities as mediators. In addition, we expect that these direct and indirect paths vary according to partnership status, as follows.

Because previous research has established that people with visual impairment are more likely to experience functional limitations, which in turn lead to depression or depressive symptomatology, we hypothesize that the link between functional limitations and depressive symptomatology is weaker among partnered than among single older adults.

Aligned with social support theory, we hypothesize that partnered older adults with vision impairment receive more emotional support than the corresponding single older adults and that emotional support is associated with less risk of depressive symptomatology in both groups.

While vision impairment may lead to loss of social relations and social interactions, the negative consequences may be alleviated by spousal support. A spouse may also facilitate social activities. We therefore hypothesize a weaker link between vision impairment and reduced participation in social activities among partnered than among single older adults.

## Method

### Procedure and participants

The analysis built on two data sources: A 2020 survey among people with vision impairment and a reference population from the most recent (2017) wave of the Danish Longitudinal Study of Ageing (DLSA). The aim of the survey among people with vision impairment was to identify and interview older adults who were limited in performing everyday tasks due to vision impairment but who were not blind. To identify older adults who potentially were eligible for our study, we drew a sample of 5000 individuals aged 55 and over from the Danish health registers among people who had (a) been diagnosed with glaucoma or age-related macular degeneration (AMD) (conditions causing vision impairment in old age), (b) visited an ophthalmologist during the preceding year and received treatment or advice related to glaucoma or AMD, or (c) been prescribed depressurizing eye drops (a treatment for glaucoma).

Respondents received information about the study via a letter in their “e-Boks”—a digital communication service connected to the individual’s national individual identifier, used by both public authorities and private firms—and were subsequently interviewed over the telephone. The Danish health registries do not include information on visual status. Therefore, potential respondents were screened according to a subset of questions from the Danish translation of the Visual Function Questionnaire (Mangione et al., 2001; Sørensen et al., 2011), and only respondents who experienced vision-related problems, but who did not characterize themselves as blind, were retained in the sample. We continued the data collection until 1,001 interviews had been conducted.^1^ In this study, we include a measure of functional limitations, which is based on a set of questions regarding inability to perform activities of daily living. These questions were only posed to respondents aged 65 and over. As information on functional limitations was not available for respondents below age 65, we excluded the 125 respondents aged 55–64.

The reference sample consisted of participants to the most recent wave of the DLSA—a representative ongoing longitudinal study covering a broad range of topics, including well-being, health, family status, social isolation and activities (Kjær et al., 2019). The 2017 wave covered individuals born every five years from 1920 to 1965 (i.e., aged 52–97 in 2017). Most of the data were collected via telephone interviews, whereas a small proportion was collected using face-to-face interviews. The reference sample is representative for the general population and therefore also includes visually impaired individuals. While the prevalence of vision impairment in the representative reference sample is unknown to us, as questions on vision impairment are not included in the DLSA, prevalence of vision impairment in Denmark has previously been estimated to be 0.6% among > 50-year-olds and 3.7% among > 80-year olds (Høeg et al., 2016). We coded respondents in the reference sample as not being visually impaired. Thus, the analysis may have underestimated the differences between visually impaired and non-impaired respondents.

We combined the data from the two samples with information from the Danish administrative population, education and income registries, which include comprehensive individual-level data on the entire population. The registries are collected and administered by Statistics Denmark and are linked at the individual level via the civil registration number.

### Measures

#### Vision impairment

The analysis included low-vision respondents who were coded as suffering from vision impairment if they met the following criteria:

1. They described their vision as “fair,” “poor” or “very poor” and reported having at least “a little difficulty” with at least one of ten everyday activities due to their vision.^2^
2. They described their vision as “good” and reported having (a) “a little difficulty” with at least three activities, (b) “a little difficulty” with at least one activity and “moderate difficulty” with at least one activity, (c) “extreme difficulty” or “stopped doing this because of my eyesight” with at least one activity or (d) worry about their eyesight “often” or “all of the time.”

Seven hundred twenty-six respondents met these criteria, with complete information on all variables in this study.^3^ In addition to individuals from this low-vision sample, the data included a reference population from the DLSA (*n* = 5.105)–coded as not visually impaired.

#### Depressive symptomatology

We assessed depressive symptomatology using the five-item World Health Organization Well-Being Index (WHO-5). The WHO-5, which measures respondents’ subjective well-being, has been validated as a screening tool for depression (Topp et al., 2015). Individuals at or above the cutoff of 13 were coded as experiencing symptoms of depression (the total score ranges from 0 to 25 with higher scores indicating more depressive symptoms, Psychiatry and Behavioral Health Learning Network, 2020).

#### Partnership status

Partnership status was based on survey information on whether the individual lived alone (coded as 0) or cohabited, i.e., lived together with a spouse or a partner (coded as 1) at the time of survey collection.

#### Functional limitations

Our indicator for functional limitations was based on a translated version of the Older American Resources and Services Multidimensional Functional Assessment Questionnaire’s section on activities of daily living (ADL) (Bushnik, 2011). It is based on questions about whether respondents are able to perform various everyday activities necessary to meeting basic physical needs (such as walking indoors and outdoors, washing and getting dressed, using the toilet and self-feeding) and instrumental activities necessary for living an independent life (such as house cleaning, taking medication, communicating by phone and preparing meals). Answers for each activity were coded 0 (= can do independently), 1 (= needs some assistance) and 2 (= requires total assistance) and summarized in a score for functional limitations ranging from 0 to 2 (Cronbach’s α = 0.89).

#### Emotional support

Perceived available emotional support was evaluated with the four questions from the short version of the Medical Outcomes Survey (mMOS-SS) (Moser et al., 2012). Questions include how often respondents have someone to have a good time with, to love or to make them feel wanted. Answers are never, rarely, sometimes, often or always. Scores were summarized and transformed to a 0–100 scale, with higher scores indicating more emotional support (Rand, 2021).

#### Social activities

Participation in social activities was a binary indicator for whether the respondent usually participates in social activities at least once a week (e.g., engaging in sports, going to the theater or the movies, playing board games or cards, or attending leisure education, senior citizens clubs or religious services).

#### Control variables

Control variables included register-based information on gender, age, education and income, and self-reported information on relative health and parental status. Gender was coded as 0 = male, 1 = female. Age was a continuous variable ranging from 65 to 97. Education was coded at two levels: low (primary, secondary or upper secondary education) and high (vocational education or short-, medium- or long-cycle higher education). Income was the natural logarithm of equivalent disposable income, measured in 2017 Danish kroner.^4^ The indicator for self-reported relative health was coded as 0 = same as most others or worse, 1 = better than most others and parental status was coded as 0 = no children or 1 = one or more children.

### Analytical strategy

To investigate the link between vision impairment and depressive symptomatology by partnership status, we merged the sample of visually impaired respondents and the reference sample (in which all respondents were coded as not being visually impaired). First, we computed descriptive statistics by partnership status and evaluated differences in distributions and means using chi-2- or t-tests, respectively. Second, using logistic analysis, we analyzed the association between vision loss and the probability of depressive symptomatology by partnership status. This second analysis confirmed the probability of depressive symptomatology depending negatively on having a partner and positively on vision impairment. Third, having established significant associations between partnership status, vision impairment and depressive symptomatology, we conducted path analysis with structural equation modeling (SEM) grouped by partnership status, to investigate *how* partnership status influences the relationship between vision impairment and depressive symptomatology. We specified separate models for partnered and single older adults, but model fit was evaluated simultaneously (multiple-group modeling approach).

The model was specified as follows: We first computed zero-order correlations between study variables (Table 2). We specified direct paths from the exogenous variable vision impairment to the four endogenous variables: depressive symptomatology functional limitations, emotional support and participation in social activities. We also specified paths from the endogenous variables functional limitations, emotional support and participation in activities to depressive symptomatology. We included sociodemographic variables (age, gender, education, income, self-rated health and child(ren)) as control variables in the model based on significant bivariate correlations. If there was a significant correlation between a mediator and a control variable, that relationship was included as a path in the model. We estimated covariances between control variables that were significantly correlated with one another. Following Bookwala & Lawson, (2011), we allowed the error variances of the mediators (functional limitations, emotional support and participation in activities) to covary.

Using Wald tests, we first investigated whether structural coefficients, structural constants and structural errors, respectively, were jointly equal across groups. Second, we applied score tests to investigate if parameters could be constrained as equal across groups (partnered and single older adults) and Wald tests to investigate whether imposed restrictions needed relaxing.

Using multiple methods, including standardized root mean squared residual (SRMR), root mean squared error of approximation (RMSEA), Tucker–Lewis index (TLI) and comparative fit index (CFI) (Hu & Bentler, 1999), we investigated the goodness of fit of the model to the data. Acceptable cutoffs for these fit indices are values of 0.08 or smaller for the SRMR, values of 0.06 or smaller for the RMSEA, values of 0.95 or larger for the TLI and values of 0.90 or larger for the CFI (Hu & Bentler, 1999). To test for between-group differences in unrestricted paths, we applied the Δz test (Kunzmann et al., 2000). We compared path coefficients rather than standardized path coefficients, as the latter may produce unreliable estimates in estimations involving multiple groups (StataCorp, 2019). To estimate and test models, we used SEM in Stata 16, with maximum likelihood estimation of path coefficients. While this estimation method formally assumes joint normality of all variables, simulations have shown that ML estimation show good results even if this assumption is violated (StataCorp, 2019).^5^

## Results

### Descriptive findings

The descriptive statistics of partnered and single older adults appear in Table 1. The percentages above the cutoff for depressive symptomatology are approximately twice as large among single (12.3%) than among partnered older adults (6.1%). The percentage with vision impairment is also slightly higher among single older adults (13.8%) than among partnered ones (11.8%). On average, single older adults receive less emotional support, have a significantly higher ADL inability score, and are significantly less likely to participate in social activities at least once a week than their partnered counterparts.

**Table 1:** Characteristics of partnered and single older adults

Characteristics	Partnered (*n* = 3908)	(SD)	Single (*n* = 1923)	(SD)	
Age (years)	72.7	(5.6)	76.5	(7.3)	***
Women (%)	45.6		69.8		***
Depressive symptomatology (%)	6.1		12.3		***
Vision impairment (%)	11.8		13.8		**
Inability to perform ADL (score)	0.1	(0.2)	0.2	(0.3)	***
Emotional support (score)	86.7	(17.0)	78.4	(22.3)	***
Participation in activities %	72.4		64.4		***
Has child(ren) %	93.9		87.4		***
Education–low %	27.7		40.3		***
Education–mid or high %	72.3		59.7		***
Good health %	43.4		47.0		**
Income (ln DKK)	12.4	(0.40)	12.3	(0.35)	***

SD: Standard deviation (reported for continuous variables). *Depressive symptomatology*—share above the cutoff for the WHO-5 screening tool for depression. *Vision impairment*—share with vision impairment as evaluated based on register data combined with screening questions. *Inability to perform ADL*–average score for inability to perform activities of daily living (range: 0–2, higher scores indicate more functional limitations). *Emotional support*–score from the short version of the Medical Outcomes Survey (mMOS-SS) (range: 0–100, higher scores indicate more emotional support). *Participation in activities*–share participating in social activities at least once a week. *Good health–*share with self-rated heath that is better than most others. *Income–*average natural logarithm of equivalent disposable income, measured in 2017 Danish kroner. ** *p* < 0.01; ***p < 0.001

Single older adults are more often female and on average slightly older than partnered older adults. Moreover, they are less likely to have child(ren) and have less education and lower incomes. Nevertheless, single older adults more often report having good health than partnered older adults.

### Findings from logistic analysis

**Fig. 1:**
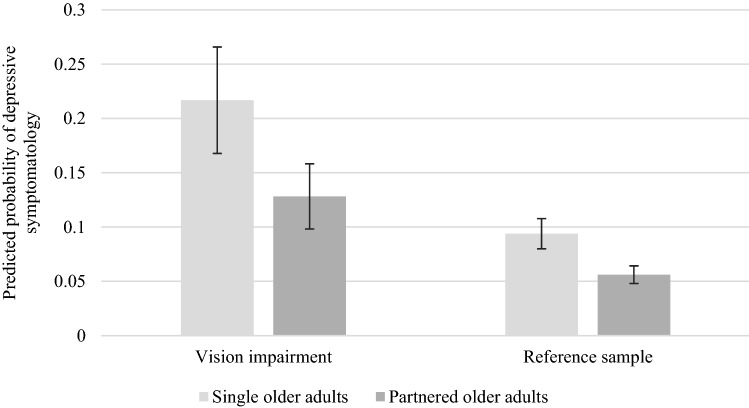
Predicted probabilities of depressive symptomatology by partnership status for people with vision impairment and reference sample. (The predicted probabilities are calculated from a logistic regression with an indicator for being above the cutoff for depressive symptomatology as the dependent variable and interaction variables between relationship status and vision impairment as explanatory variables. The regression controls for age, gender, education, income, parental status and relative self-reported health. Vertical lines show confidence intervals at the 95% level. We calculate standard errors using the delta method).

Figure 1 presents predicted probabilities of the risk of depressive symptomatology from a logistic regression model, including interactions between vision impairment and partnership status as explanatory variables.

As expected, the figure shows significant associations between both vision impairment and relationship status and the risk for depressive symptomatology. The risk for depressive symptomatology is nearly four times greater among people with vision impairment who live alone than among people in the reference sample who cohabit. Thus the interaction between vision impairment and partnership status is strongly associated with depressive symptomatology.

### Findings from path analysis

Table 2 shows zero-order correlations between study variables for partnered and single older adults. As expected, vision impairment is significantly correlated with more functional limitations, a lower probability of participation in social activities, and a higher probability of depression in both groups. However, while vision impairment (as expected) is positively correlated with more emotional support among single older adults, contrary to our expectation this correlation is negative among partnered older adults. This difference in the direction of correlations provides indicative evidence of the presence of group differences in paths.

**Table 2:** Bivariate correlation between study variables by partnership status

Single older adults	(2)	(3)	(4)	(5)	(6)	(7)	(8)	(9)	(10)	(11)
Vision impairment (1)	0.13***	0.12***	0.07***	− 0.05*	0.14***	0.07***	0.08***	− 0.02	− 0.01	0.03
Risk of depression (2)		0.31***	− 0.22***	− 0.19***	− 0.02	0.01	− 0.17***	− 0.03	− 0.06*	− 0.01
Functional limitations (3)			− 0.19***	− 0.32***	0.38***	0.04	− 0.19***	− 0.19***	− 0.13***	0.03
Emotional support (4)				0.22***	− 0 − 03	0.15***	0.11***	0.06***	0.08***	0.16***
Social activities (5)					− 0.06***	0.10***	0.13***	0.15***	0.14***	0.02
Age (6)						0.12***	0.18***	− 0.18***	− 0.14***	0.17***
Gender (7)							0.02	− 0.02	− 0.07***	0.13***
SRH (8)								0.04*	0.07***	0.06**
Education (9)									0.35***	− 0.05**
Income (10)										− 0.05**
Children (11)										
Partnered older adults	(2)	(3)	(4)	(5)	(6)	(7)	(8)	(9)	(10)	(11)
Vision impairment (1)	0.09***	0.19***	− 0.05***	− 0.08***	0.16***	0.00	0.01	− 0.00	− 0.06***	− 0.01
Risk of depression (2)		0.26***	− 0.12***	− 0.13***	0.01	0.07***	− 0.16***	− 0.10***	− 0.14***	− 0.02
Functional limitations (3)			− 0.11***	− 0.21***	0.25***	0.07***	− 0.16***	− 0,10***	− 0.14***	− 0.01
Emotional support (4)				0.09***	− 0.06***	0.08***	0.03*	0.05**	0.08***	0.06***
Social activities (5)					− 0.04*	0.03*	0.10***	0.13***	0.13***	0.04*
Age (6)						− 0.07***	0.12***	− 0.11***	− 0.39***	0.05***
Gender (7)							− 0.10***	− 0.01	− 0.05***	0.03*
SRH (8)								0.04***	0.04***	0.02*
Education (9)									0.33***	− 0.01
Income (10)										− 0.02
Children (11)										

Higher scores represent suffering from visual impairment, being above the threshold for the WHO-5 screening tool for depression, more functional limitations, more emotional support, participating in social activities at least once a week, being older, being female, having self-rated heath (SRH) that is better than most others, having vocational education or short-, medium- or long-cycle higher education, having a higher income, having at least one child. *p < 0.05, ** *p* < 0.01; ***p < 0.001

To test our hypotheses, we conducted a path analysis in which we hypothesized that the path between poor vision and depressive symptomatology is mediated by functional limitations, emotional support and participation in activities—and that these paths vary by relationship status. As Table 3 shows, this initial model (M1) had good fit.

**Table 3:** Model fit indices

	Chi-2	*df*	*P*	CFI	TLI	RMSEA	SRMR	ΔChi-2	Δdf	*p*
M1: Unconstrained model	10.330	4	0.035	0.998	0.958	0.023	0.005			
M2: Constrained model, paths restricted to equality across groups	43.094	31	0.073	0.995	0.990	0.012	0.011	32.764	27	0.205
M3: Constrained model, insignificant paths restricted to 0	43.953	34	0.118	0.996	0.992	0.010	0.011	33.623	30	0.296

Cutoff values are > 0.90 for comparative fit index (CFI); > 0.95 for Tucker–Lewis index (TLI); < 0.06 for root mean squared error of approximation (RMSEA), and < 0.08 for standardized root mean squared residual (SRMR)

Second, we used Wald tests to investigate whether structural coefficients, structural constants and structural errors, respectively, were jointly equal across groups (partnered and single older adults). These tests revealed that each of the three hypotheses of joint equality could be rejected. These results support our hypothesis that the association between vision impairment and depressive symptomatology differs according to partnership status.

Wald and score tests revealed that the following three paths did not differ across groups (partnered and single older adults): the path from vision impairment to functional limitations, the path from vision impairment to participation in activities, and the path from participation in activities to depressive symptomatology. The constrained model (M2) also had good fit and did not differ significantly from the unconstrained model (M1) (Table 3).

**Fig. 2:**
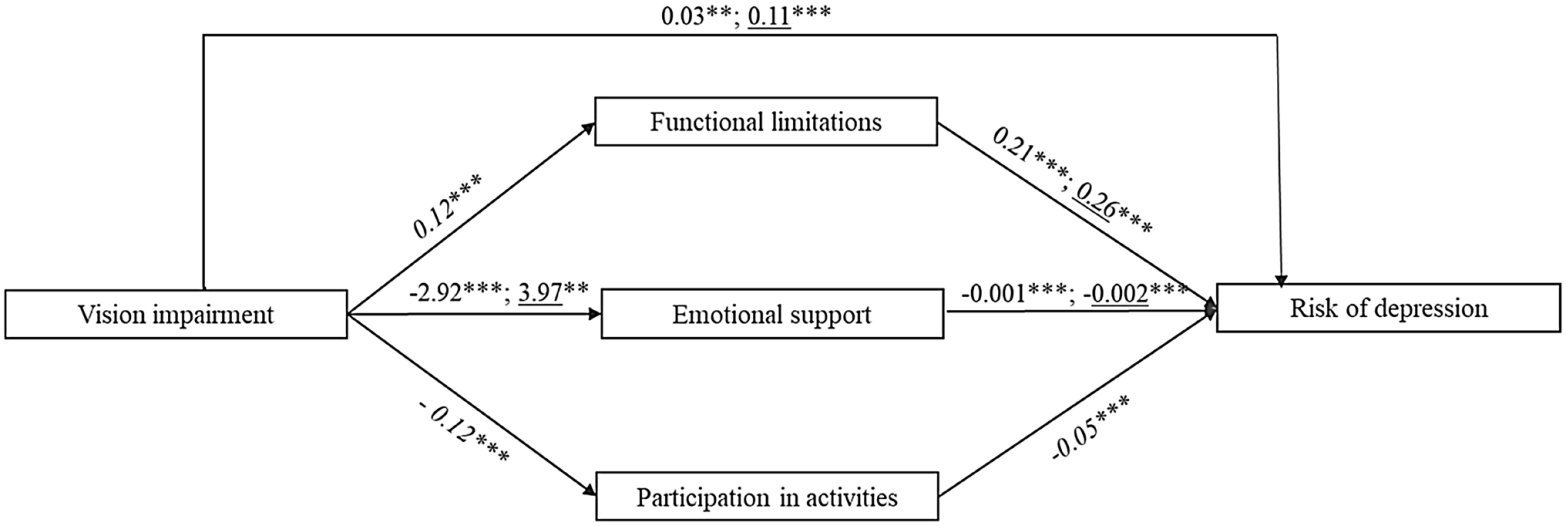
Illustration of final path model, testing functional limitations, emotional support and participation in activities as mediators of the link between vision impairment and risk of depression by partnership status. (While correlations between outcomes were allowed, they are omitted from the figure for reasons of clarity. Path coefficients of partnered individuals; path coefficients of single individuals; *path coefficients restricted to be equal across groups.* ** *p* < 0.01; ***p < 0.001).

Third, we constrained insignificant paths as equal to 0 (only a few paths from control variables to endogenous variables were insignificant). Again, this model (M3) had good fit and did not differ significantly from the first model (Table 3). Figure 2 presents regression coefficients for the key paths of the final model (M3).

As we hypothesized, a direct link exists between vision impairment and depressive symptomatology, regardless of partnership status. This direct link is significantly larger among older adults without a partner than among older adults with one (*β*_s_ = 0.11, *β*_p_ = 0.03, Δz = -4.72, *p* < 0.01). Moreover, regardless of partnership status, the link between vision impairment and depressive symptomatology is mediated by functional limitations, emotional support and participation in activities.

We find that the link between vision impairment and functional limitations does not differ by partnership status (*β* = 0.12, restricted to equality across groups based on results from score and Wald tests). However, as we hypothesized, the relationship between functional limitations and depressive symptomatology is stronger among those living alone than among partnered individuals (*β*_s_ = 0.26, *β*_p_ = 0.21, Δz = −2.47, *p* < 0.01). This result is consistent with our hypothesis that a partner can assist in performing ADLs and that receiving such assistance attenuates the link between ADL inability and depression among partnered older adults.

Although the link between vision impairment and depressive symptomatology is mediated by emotional support, this link is negative for people who are partnered, but positive for people who live alone (*β*_s_ = 3.97, *β*_p_ = −2.92, Δz = −5.96, *p* < 0.01). The negative link for partnered older adults is contrary to our expectation. Nonetheless, as we expected, emotional support is associated with less depression, regardless of partnership status. This association is stronger among single older adults than among partnered ones (*β*_s_ = −0.002, *β*_p_ = −0.001, Δz = 2.94, *p* < 0.01).

Contrary to our hypothesis, the link between vision impairment and the probability of participating regularly in social activities does not vary according to partnership status (*β* = −0.12, restricted to equality across groups based on the results from score and Wald tests). Moreover, while as expected people who participate regularly in social activities have a lower probability of depressive symptomatology, we find no difference by partnership status in this link (*β* = −0.05, restricted to equality across groups based on the results from score and Wald tests).

A test for overall indirect effects of vision impairment on depressive symptomatology reveals that the three mediators have significant, albeit small, indirect impact regardless of partnership status. The collective indirect effect of the mediators does not vary by partnership status (*β*_s_ = 0.03, *β*_p_ = 0.03, Δz = 1.13, n.s.). Thus the differences between partnered and single older adults in the association between vision impairment and depressive symptomatology can be attributed mainly to the much stronger direct link from vision impairment to the risk of depression among single older adults. In addition, that the direct link from vision impairment to emotional support is negative among partnered older adults, but positive among single ones, constitutes an important difference between the two groups.

## Discussion

The aim of this study was (a) to investigate separately by partnership status the direct link from vision impairment to depressive symptomatology and (b) to determine whether this link was mediated by functional limitations, emotional support and participation in activities. The analysis reveals that the data are consistent with our suggested model and that some of the paths we investigated vary according to partnership status.

We find that vision impairment predicts a higher risk of depressive symptomatology, regardless of partnership status. However, this link is more than twice as large among single older adults than among partnered ones. This finding indicates that having a partner significantly reduces the negative association between vision impairment and depressive symptomatology—regardless of relationship quality (Bookwala, 2011).

Moreover, the link between vision impairment and functional limitations does not differ by partnership status. This result is not surprising, as having a partner does not per se affect the ability of people with vision impairment to individually perform activities of daily living. However, having a partner weakens the link between functional limitations and depressive symptomatology among people with vision impairment. We interpret this finding as support for our hypothesis that a partner can help such a person perform activities of daily living, thereby reducing the risk of depression among partnered older adults with vision loss.

Contrary to our hypothesis, partnered older adults with vision loss perceive having *less* emotional support than partnered older adults in general, whereas single older adults with vision loss perceive having *more* emotional support than single older adults in general. Previous studies have indicated that impairments may lead to an intensification in the availability of various forms of social support (Carr et al., 2019; Kempen et al., 2012), but we find this only for our single respondents with vision impairment. Loss of vision is distressing, not only for the individuals suffering from it, but also for their spouses (Lehane et al., 2017; Strawbridge et al., 2007). Thus, due to negative spillover effects of their partner’s impairment on their own mental well-being, the partners of people with vision loss may be less able to provide emotional support. In addition, single older adults may compensate for the lack of a partner by increasing other social ties and potential sources of support (Sarkisian & Gerstel, 2015) and, consequently, receive more emotional support than their partnered counterparts (Stokes & Moorman, 2018).

Although emotional support reduces the risk for depression of both partnered and single older adults, this link is stronger among single ones, that is, single older adults obtain better buffering effects from emotional support in terms of depressive symptomatology than partnered older adults do. One possible explanation is that a partner can provide various types of support (e.g., social, emotional, affectionate, tangible or financial), and an increase or decrease in one type of support (in this instance, emotional support) may therefore have a smaller effect on the overall well-being of partnered individuals.

Contrary to our expectations, no group differences emerged for the path between vision impairment and participation in activities. Thus although a partner may facilitate a visually impaired person’s participation in activities, our results do not support the existence of such a link. Older adults with vision loss may lose interest or enjoyment in social activities simply because these activities require more effort for them (O’Donnell, 2005). Such disengagement may explain why the probability of participation does not vary by partnership status: If older adults with vision impairment lose interest in participating in social activities, having a partner to assist with participation has no significance.

### Limitations and future research

This study has four potential limitations that need considering when interpreting the findings. First, it defines vision impairment based on medical register data supplemented with respondents’ accounts of experiences of limitations in day-to-day activities due to vision loss. This subjective definition of vision impairment is a potential limitation as discrepancies may exist between subjective and objective measures of vision impairment. However, previous comparisons of self-rated vision to clinical measures found that better self-rated vision was associated with, for example, better visual acuity, contrast sensitivity, stereoacuity and visual fields (El-Gasim et al., 2013; Whillans & Nazroo, 2014). In addition, studies have argued that a self-reported measure might more accurately reflect the wider aspects of visual functioning than objective measures (Matthews et al., 2017). Moreover, research has suggested that self-reported vision is more strongly associated with mental well-being than objective measures (Bookwala & Lawson, 2011). As we do not have access to an objective measure of vision, we cannot investigate possible differences between the two types of measures when investigating the link between vision impairment and depressive symptomatology by partnership status. Moreover, by assuming that no one in the reference sample has a vision impairment, we may underestimate the link between vision impairment and depressive symptomatology. However, as this potential bias will affect partnered and single older adults in the same way, we do not expect it to influence the differences in coefficients between groups.

Second, our measure of partnership was based on cohabitation, i.e., whether or not the respondent lived together with a partner or not. While defining partnership status based on cohabitation may give a more reliable indication of a steady availability of a partner, we cannot rule out the possibility that by not differentiating between single-living persons with and without a partner we miss some important information. According to a previous study, the share of persons “living apart together” is five percent in the Danish population aged 52 and older (Siren & Larsen, 2019).

Third, due to a limited sample size, this study cannot establish whether the link between vision impairment and functional limitations varies systematically by other variables, such as gender, age or personality traits, in addition to partnership status. Previous findings indicate some gender differences in that while husbands with health problems receive substantial emotional and practical caregiving from wives, the reverse may not be the case (Wong & Waite, 2015). Due to gradual adaptation to age-related loss of functioning and visual capacity, the link between vision loss and depressive symptomatology by partnership status, may vary with age. Jopp et al., (2008) show that good visual capacity is among the most important contributors to Valuation of Life among young-olds (65–79 years of age), whereas no significant association appears among old-olds (80–94 years of age). Finally, some previous research (Wahl et al., 2012) show that older adults’ personality traits may influence how well they cope with visual impairment and the inclusion of personality measures may have provided additional insights as regards path differences according to partnership status between vision impairment and depressive symptomatology.

Finally, as we only have access to cross-sectional data, we cannot investigate the causal relationship between variables. Taken together, performing analyses of the link between vision impairment and depressive symptomatology separately by gender or age-group, as well as by partnership status are important topics for future research.

## Conclusions

Our results suggest that partnered older adults are less distressed by vision impairment than single ones. However, our results also indicate that single older adults with vision impairment actually perceive having more emotional support than their partnered counterparts. Consequently, single older adults may—at least somewhat—compensate for the lack of a partner by establishing close relationships with other people, and our result suggests that single older adults with vision impairment may be better at reaching out to peers, other family members, and other help than partnered ones, and/or that surrounding sources of social support may acknowledge their needs more often. In addition, a visual impairment in one partner may have negative consequences on the mental health of the other. In such cases, the partner’s ability to provide both practical and emotional support may be compromised. Therefore, considering not only partnership status but also the mental health of both spouses is crucial when targeting interventions aimed at reducing the risk of depression in older adults with vision impairment.

## Footnotes

1 The data used in this study was originally collected for a commissioned report that aimed at descriptively comparing people with and without vision impairment. The sample size for that report (1.000 respondents) was determined based on previous experiences of conducting similar research, but also for pragmatic and financial reasons.

2 The activities included reading ordinary print in newspapers; doing work or hobbies that require one to see well up close (such as cooking, sewing, fixing things around the house, or using hand tools); finding something on a crowded shelf; reading street signs or the names of stores; going down steps, stairs, or curbs in dim light or at night; noticing objects off to the side while walking; seeing how people react to things one say; picking out and matching one’s clothes; visiting with people in their homes, at parties, or in restaurants; going out to see movies, plays, or sports events.

3 Our sample consisted of 876 respondents above the age of 65. We excluded 73 respondents due to not living up to our criteria for vision impairment and a further 75 respondents due to incomplete information on ability to perform activities of daily living (43 respondents) or self-reported relative health (33 respondents) (one respondent had missing information on both variables). Respondents with missing information on any of the variables were excluded from all analyses.

4 1 EUR ≈ 7.4 Danish kroner. We use the natural logarithm of income to avoid scale-differences in coefficients.

5 Violation of the normality assumption has been found to primarily influence log-likelihood and chi-2 tests (StataCorp, 2019). As our analysis relied on other types of tests to investigate if parameters could be constrained to be equal across groups, and to evaluate the goodness-of-fit of the model to the data, we argue that our estimates are reliable.

## References

[CR1] AARP and National Alliance for Caregiving. Caregiving in the United States 2020. Washington, DC: AARP. May 2020. 10.26419/ppi.00103.001

[CR2] Alma MA, Van Der Mei SF, Feitsma WN, Groothoff JW, Van Tilburg TG, Suurmeijer TPBM (2011). Loneliness and self-management abilities in the visually impaired elderly. J Aging Health.

[CR3] Bookwala J (2011). Marital quality as a moderator of the effects of poor vision on quality of life among older adults. J Gerontol–series B Psychol Sci Soc Sci.

[CR4] Bookwala J, Lawson B (2011). Poor vision, functioning, and depressive symptoms: a test of the activity restriction model. Gerontologist.

[CR5] Bourne RRA, Flaxman SR, Braithwaite T, Cicinelli MV, Das A, Jonas JB, Keeffe J, Kempen J, Leasher J, Limburg H, Naidoo K, Pesudovs K, Resnikoff S, Silvester A, Stevens GA, Tahhan N, Wong T, Taylor HR, Ackland P, Zheng Y (2017). Magnitude, temporal trends, and projections of the global prevalence of blindness and distance and near vision impairment: a systematic review and meta-analysis. Lancet Glob Health.

[CR6] Brown SL, Wright MR (2017). Marriage, Cohabitation, and Divorce in Later Life. Innov Aging.

[CR7] Bushnik, T. (2011). Older Americans’ Resources and Services Multidimensional Functional Assessment Questionnaire. In Encyclopedia of Clinical Neuropsychology (pp. 1811–1812). Springer New York.

[CR8] Carr D, Cornman JC, Freedman VA (2019). Do family relationships buffer the impact of disability on older adults’ daily mood? An exploration of gender and marital status differences. J Marriage Fam.

[CR9] Carrière I, Delcourt C, Daien V, Pérés K, Féart C, Berr C, Ancelin ML, Ritchiea K (2013). A prospective study of the hi-directional association between vision loss and depression in the elderly. J Affect Disord.

[CR10] Cohen S (2004). Social relationships and health. In Am Psychol.

[CR11] Cosh S, Carriere I, Nael V, Tzourio C, Delcourt C, Helmer C (2019). The association of vision loss and dimensions of depression over 12 years in older adults: findings from the three city study. J Affect Disord.

[CR49] Dykstra PA, Hagestad GO (2007) Childnessness and parenthooed in two centuries: different roads - different maps? J Family Issues 28(11):1518–1532. 10.1177/0192513X07303881

[CR12] El-Gasim M, Munoz B, West SK, Scott AW (2013). Associations between self-rated vision score, vision tests, and self-reported visual function in the salisbury eye evaluation study. Invest Ophthalmol vis Sci.

[CR13] Evans JR, Fletcher AE, Wormald RPL (2007). Depression and anxiety in visually impaired older people. Ophthalmology.

[CR14] Gong X, Ni Z, Wu B (2020). The mediating roles of functional limitations and social support on the relationship between vision impairment and depressive symptoms in older adults. Ageing Soc.

[CR15] Gopinath B, Liew G, Burlutsky G, Mitchell P (2014). Age-related macular degeneration and 5-year incidence of impaired activities of daily living. Maturitas.

[CR16] Hajek A, Brettschneider C, Eisele M (2016). Effect of visual impairment on physical and cognitive function in old age: findings of a population-based prospective cohort study in germany. J Am Geriatr Soc.

[CR17] Hochberg C, Maul E, Chan ES, Van Landingham S, Ferrucci L, Friedman DS, Ramulu PY (2012). Association of vision loss in glaucoma and age-related macular degeneration with IADL disability. Invest Ophthalmol vis Sci.

[CR18] Hu LT, Bentler PM (1999). Cutoff criteria for fit indexes in covariance structure analysis: conventional criteria versus new alternatives. Struct Equ Model.

[CR19] Jopp D, Rott C, Oswald F (2008). Valuation of life in old and very old age: the role of sociodemographic, social, and health resources for positive adaptation. Gerontologist.

[CR20] Kempen GIJM, Ballemans J, Adelita R, V., Van Rens, G. H. M. B., & Zijlstra, G. A. R.  (2012). The impact of low vision on activities of daily living, symptoms of depression, feelings of anxiety and social support in community-living older adults seeking vision rehabilitation services. Qual Life Res.

[CR21] Kempen GIJM, Ranchor AV, Ambergen T, Zijlstra GAR (2014). The mediating role of disability and social support in the association between low vision and depressive symptoms in older adults. Qual Life Res.

[CR22] MH Kim RE Dunkle AJ Lehning H-W Shen S Feld AK Perone 2016 Caregiver Stressors and Depressive Symptoms among Older Husbands and Wives in the United States 10.1080/08952841.2016.1223962 10.1080/08952841.2016.1223962 27673406

[CR23] Kjær AA, Siren A, Seestedt MH, Fridberg T, Casier F (2019). Cohort profile: the danish longitudinal study of ageing (DLSA). Int J Epidemiol.

[CR24] Kunzmann U, Little TD, Smith J (2000). Is age-related stability of subjective well-being a paradox? Cross-sectional and longitudinal evidence from the Berlin aging study. Psychol Aging.

[CR25] Lehane CM, Dammeyer J, Elsass P (2017). Sensory loss and its consequences for couples’ psychosocial and relational wellbeing: an integrative review. In Aging and Mental Health.

[CR26] Lehane CM, Elsass P, Hovaldt HB, Dammeyer J (2018). A relationship-focused investigation of spousal psychological adjustment to dualsensory loss. Aging Ment Health.

[CR27] Lehane CM, Hofsöe SM, Wittich W, Dammeyer J (2018). Mental health and spouse support among older couples living with sensory loss. J Aging Health.

[CR28] Mangione CM, Lee PP, Gutierrez PR, Spritzer K, Berry S, Hays RD (2001). Development of the 25-item national eye institute visual function questionnaire. Arch Ophthalmol.

[CR29] Matthews K, Nazroo J, Whillans J (2017). The consequences of self-reported vision change in later-life: evidence from the english longitudinal study of ageing. Public Health.

[CR30] Moser A, Stuck AE, Silliman RA, Ganz PA, Clough-Gorr KM (2012). The eight-item modified medical outcomes study social support survey: psychometric evaluation showed excellent performance. J Clin Epidemiol.

[CR31] O’Donnell, C. (2005). The Greatest Generation Meets Its Greatest Challenge: Vision Loss and Depression in Older Adults-Aging-April 2005 The Greatest Generation Meets Its Greatest Challenge: Vision Loss and Depression in Older Adults. *Journal of Visiual Impairment and Blindness*, *99*(4). http://www.afb.org/jvib/jvib990402.asp

[CR32] Psychiatry & Behavioral Health Learning Network. (2020). *WHO (Five) Well-Being Index (WHO-5)*. https://www.psychcongress.com/saundras-corner/scales-screeners/well-being-index/who-five-well-being-index-who-5

[CR33] Rand. (2021, February 2). *Social Support Survey Instrument Scoring Instructions | RAND*. https://www.rand.org/health-care/surveys_tools/mos/social-support/scoring.html

[CR34] Sarkisian N, Gerstel N (2015). Does singlehood isolate or integrate? Examining the link between marital status and ties to kin, friends, and neighbors. J Soc Pers Relat.

[CR48] Siren A, Larsen MR (2019) Ældres familiære og sociale relationer. Analyser på baggrund af Ældredatabasens 5. bølge. VIVE - Det Nationale Forsknings- og Analysecenter for Velfærd, Copenhagen

[CR35] Siren A, Casier F, Amilon A (2018). Barnløshed og familieform i det sene voksenliv: Sammenligning af ældre barnløse og forældre i forhold til socioøkonomiske karakteristika, hverdagsliv og velbefindende.

[CR36] Sørensen MS, Andersen S, Henningsen GØ, Larsen CT, Sørensen TL (2011). Danish version of visual function questionnaire-25 and its use in age-related macular degeneration. Dan Med Bull.

[CR37] StataCorp. (2019). *Stata Structural Equation Modeling Reference Manual - Release 16*.

[CR38] Stevens GA, White RA, Flaxman SR, Price H, Jonas JB, Keeffe J, Leasher J, Naidoo K, Pesudovs K, Resnikoff S, Taylor H, Bourne RRA (2013). Global prevalence of vision impairment and blindness: magnitude and temporal trends, 1990–2010. Ophthalmology.

[CR39] Stokes JE, Moorman SM (2018). Influence of the social network on married and unmarried older adults’ mental health. Gerontologist.

[CR40] Strawbridge WJ, Wallhagen MI, Shema SJ (2007). Impact of spouse vision impairment on partner health and well-being: a longitudinal analysis of couples. J Gerontol–series B Psychol Sci Soc Sci.

[CR41] Taylor DJ, Hobby AE, Binns AM, Crabb DP (2016). How does age-related macular degeneration affect real-world visual ability and quality of life? A systematic review. BMJ Open.

[CR42] Taylor DJ, Jones L, Binns AM, Crabb DP (2020). ‘You’ve got dry macular degeneration, end of story’: a qualitative study into the experience of living with non-neovascular age-related macular degeneration. Eye (basingstoke).

[CR43] Topp CW, Dinesen Østergaard S, Søndergaard S, Bech P (2015). EThe WHO-5 Well-Being Index: a Systematic Review of the Literature. Psychother Psychosom.

[CR44] van Nispen RMA, Vreeken HL, Comijs HC, Deeg DJH, van Rens GHMB (2016). Role of vision loss, functional limitations and the supporting network in depression in a general population. Acta Ophthalmol.

[CR45] Wahl HW, Heyl V, Schilling O (2012). Robustness of personality and affect relations under chronic conditions: The case of age-related vision and hearing impairment. J Gerontol–series B Psychol Sci Soc Sci.

[CR46] Whillans J, Nazroo J (2014). Assessment of visual impairment: the relationship between self-reported vision and “gold-standard” measured visual acuity. Br J vis Impair.

[CR47] Wong JS, Waite LJ (2015). Marriage, social networks, and health at older ages. J Population Ageing.

